# Chemometric Discrimination of the Geographical Origin of Three Greek Cultivars of Olive Oils by Stable Isotope Ratio Analysis

**DOI:** 10.3390/foods10020336

**Published:** 2021-02-04

**Authors:** Maria Tarapoulouzi, Vasiliki Skiada, Sofia Agriopoulou, David Psomiadis, Catherine Rébufa, Sevastianos Roussos, Charis R. Theocharis, Panagiotis Katsaris, Theodoros Varzakas

**Affiliations:** 1 Department of Chemistry, Faculty of Pure and Applied Science, University of Cyprus, P.O. Box 20537, CY-1678 Nicosia, Cyprus; tarapoulouzi.maria@ucy.ac.cy (M.T.); charis@ucy.ac.cy (C.R.T.); 2 Department of Food Science and Technology, Faculty of Agriculture and Food, University of the Peloponnese, Antikalamos, 24100 Kalamata, Greece; vpskiada@yahoo.gr (V.S.); sagriopoulou@gmail.com (S.A.); 3 Department of Olive and Horticultural Plants, Hellenic Agricultural Organization—DEMETER, 24100 Kalamata, Greece; pankatsaris@yahoo.gr; 4 Imprint Analytics GmbH, Werner von Siemens Straße 1, 7343 Neutal, Austria; psomiadis@imprint-analytics.at; 5 Aix Marseille Univ, Avignon Université, CNRS, IRD, IMBE, Campus Saint Jerome, 13013 Marseille, France; c.rebufa@univ-amu.fr (C.R.); sevastianos.roussos@imbe.fr (S.R.)

**Keywords:** stable isotope ratios, Greek olive oils, chemometric analysis, OPLS-DA, discrimination, fraud, authenticity, adulteration, geographical origin, quality

## Abstract

Α stable isotope ratio mass spectrometer was used for stable isotope ratio (i.e., δ^13^C, δ^18^O, and δ^2^H) measurements, achieving geographical discrimination using orthogonal projections to latent structures discriminant analysis. A total of 100 Greek monovarietal olive oil samples from three different olive cultivars (cv. Koroneiki, cv. Lianolia Kerkyras, and cv. Maurolia), derived from Central Greece and Peloponnese, were collected during the 2019–2020 harvest year aiming to investigate the effect of botanical and geographical origin on their discrimination through isotopic data. The selection of these samples was made from traditionally olive-growing areas in which no significant research has been done so far. Samples were discriminated mainly by olive cultivar and, partially, by geographical origin, which is congruent with other authors. Based on this model, correct recognition of 93.75% in the training samples and correct prediction of 100% in the test set were achieved. The overall correct classification of the model was 91%. The predictability based on the externally validated method of discrimination was good (Q^2^ (cum) = 0.681) and illustrated that δ^18^O and δ^2^H were the most important isotope markers for the discrimination of olive oil samples. The authenticity of olive oil based on the examined olive varieties can be determined using this technique.

## 1. Introduction

Olive oil plays an important role in the diet in Greece as well as in other Mediterranean countries. The various health benefits of olive oil consumption are well known, increasing its reputation [1,2,3,4,5]. The high price of extra virgin olive oil makes it susceptible to fraudulent activities. For instance, deliberate mislabeling of lower commercial-grade olive oils or even mislabeling by a false declaration of origin may occur [6]. For these reasons, a series of criteria and standards associated with the genetic variety, the geographical origin, and the quality grade have been established by the European Union (EU) so as to offer both fair trade in the olive sector as well as safety and protection guarantees for consumers [7,8]. Two of the most well-known denominations related to foodstuff authenticity are the Protected Designation of Origin (PDO) and the Protected Geographical Indication (PGI) [9,10,11,12,13].

The continuous evaluation of olive oil is a key priority as it holds great importance in the Mediterranean diet. Recently, specific attention has been focused on geographical and cultivar traceability by using several advanced analytical techniques, for instance, proton-nuclear magnetic resonance [14], liquid chromatography [15], luminescence [16], Fourier transform infrared spectroscopy [17], near-and- mid-infrared spectroscopy [18,19,20], as well as triacylglycerol, fatty acid, squalene, and tocopherol determinations [19,21,22,23].

Simple and efficient cultivar and geographical discrimination are of crucial importance to judge labeling compliance. Agricultural practices applied in specific areas, as well as geoclimatic characteristics, are reflected in the content of stable isotopes of bioelements (H, C, N, O, and S) and determined experimentally with isotope ratio mass spectrometry (IRMS) [24]. The IRMS technique is a well-established method for determining the authenticity of olive oil [14,25,26,27,28,29]. The stable isotope ratios of ^13^C/^12^C, ^18^O/^16^O, and ^2^H/^1^H give many promises for proof of food authenticity, as this analytical technique offers more accuracy and sensitivity compared to other less expensive and equally effective techniques [30]. Latitude and altitude, distance from the sea, and environmental and climate-related conditions are some of the factors that influence ^13^C/^12^C, ^18^O/^16^O, and ^2^H/^1^H ratios [31].

Although indigenous Greek monocultivar olive oils are more than 40 [32], with cv. Koroneiki being the most systematically cultivated variety, detailed investigation has not been carried out for local autochthonous monocultivars. For example, the local variety of Lianolia Kerkyras is cultivated exclusively on the coasts of northwestern Greece, while cv. Maurolia, which according to our knowledge has never before been characterized, is cultivated exclusively in northern Peloponnese, and more precisely in a small area of the regional unit of Messinia [33,34]. Hence, the combination of the cultivar species (cv. Koroneiki, cv. Lianolia Kerkyras, and cv. Maurolia) that have been chosen for study have only been partly evaluated for genotyping and molecular characterization [35,36], as well as for antioxidant content [1,37] and chemical composition [38].

Chemometrics and discriminant analysis are important tools for food authenticity. The supervised method orthogonal projections to latent structures discriminant analysis (OPLS-DA) is very useful for the discrimination of samples in food analysis [39,40,41,42,43,44]. Recently, OPLS-DA has been used extensively by several authors in the olive oil authentication field with great success [15,45,46,47,48,49].

The first goal of this research study was the achievement of a preliminary discrimination of the above-mentioned Greek monocultivar olive oils according to olive cultivar by applying a multivariate analysis to the IRMS measurements. The second goal was to produce a robust model capable of identifying any adulteration of unknown olive oils in Greece regarding cultivar and geographical origin. One of the most innovative points of this study was that the examined three monocultivar olive oils (cv. Koroneiki, cv. Lianolia Kerkyras, and cv. Maurolia) had never before been evaluated by IRMS in combination with chemometrics. By measuring the O, H, and C isotope ratios, the goal of this study has been successfully reached. Chemometric analysis with combination of SIMCA software was used to interpret the measurements. The OPLS-DA chemometric method was implemented. External validation was achieved by dividing the dataset into two groups. The first group (training set) was used for the adjustment of the model parameters and the second group (test set) was used for the estimation of generalization error [50].

## 2. Materials and Methods

### 2.1. Sampling

A set of 100 monovarietal olive oil samples were gathered from various olive mills in Central Greece and Peloponnese, located in different geographical areas. Particularly, 38, 29, and 33 samples of the three cultivars cv. Maurolia from Messinia (37.25° N, 21.95° E) and both cv. Lianolia Kerkyras and cv. Koroneiki from Preveza (38.95° N, 20.75° E) were collected, respectively. All samples were derived from the 2019–2020 harvesting period. This was a significant factor in the evaluation of core geographical information from the dataset in order to avoid differences due to seasonal effects. Additionally, long storage effects of olive oils (e.g., through oxidation) had to be eliminated [51]. To this end, only fresh, recently harvested olive oils were used in this study. Since stable isotope ratios are related to parameters like latitude, mean annual temperature, and average relative humidity at the collection area, the samples were collected from coastal areas, i.e., Messinia and Preveza.

### 2.2. IRMS Analysis

Stable isotope ratios of carbon (^13^C/^12^C) were measured using a horizon isotope ratio mass spectrometer (Nu Instruments Limited, Wrexham, UK) following total combustion in a Euro EA-CHNSO 2 dual elemental analyzer (EuroEA3000, EuroVector Srl, Pavia, Italy). Stable isotope ratios of oxygen and hydrogen (^18^O/^16^O, ^2^H/^1^H) were measured with a HTCEA (high temperature conversion elemental analyzer, Hekatech Gmbh, Wegberg, Germany) connected to a horizon isotope ratio mass spectrometer (Nu Instruments Limited, Wrexham, UK). Approximately 1 mL sample volume was used for each measurement.

The measured isotope ratios of each sample were normalized to a pulse of respective reference gases (CO_2_, CO, H_2_). Every sample was measured in duplicate. Each measurement sequence included two reference materials with known isotope signatures (2-point referencing) and multiple quality control samples to monitor sequence precision and accuracy.

Carbon, hydrogen, and oxygen stable isotope ratio analysis was performed at Imprint Analytics GmbH (Neutal, Austria) following the requirements of EN ISO17025:2018 accreditation standard. The method validation reported repeatability of 0.2‰, 1.9‰, and 0.2‰ for carbon, hydrogen, and oxygen isotope ratios, respectively. The accuracy of the reference materials (international reference materials, inhouse reference materials, and quality control samples) was controlled by the SD of replicate analysis during the runs, as well as quality control charts, and was under 0.1‰, 1.3‰, and 0.1‰ for carbon, hydrogen, and oxygen isotope ratios, respectively.

The values were denoted in delta (δ) in relation to the international VPDB (Vienna-Pee Dee Belemnite) and VSMOW (Vienna-Standard Mean Ocean Water) standards for δ^13^C, δ^18^O, and δ^2^H, respectively, according to the following general equation:

δ^13^Cs = (R_s_/R_std_) − 1 (1)

where R is the ^13^C/^12^C ratio of the sample (s) and of the standard (std) [52] (similarly for ^18^O/^16^O and ^2^H/^1^H) expressed in per mil (‰).

### 2.3. Statistical Analysis

SIMCA version 15.0.2 (Umetrics, Umeå, 907 29, Sweden) was used for multivariate statistical analysis. Initially, by applying chemometrics, the unsupervised (principal component analysis) PCA method was performed (not shown here). The supervised OPLS-DA procedure was then applied to discriminate and classify the olive oil samples. OPLS-DA was applied to distinguish the wrongly classified samples and to test the robustness of the model. Scaling to unit variance (UV) and mean-centering were the two settings before chemometric analysis. The application of classification methods was done after dividing the olive oil samples into three classes: cv. Maurolia (Messinia): class 1, cv. Lianolia Kerkyras (Preveza): class 2, and cv. Koroneiki (Preveza): class 3.

The 100-sample dataset was divided into training and test sets to apply external validation. Additionally, 80 and 20 samples were randomly selected to represent the training and the test set, respectively. The symbol A was used for the number of important selected components. Hotelling’s T2 confidence region is defined by the ellipse in the score scatter plots, providing a multivariate generalization of Student’s *t-*test, and a 95% confidence interval for the observations. Determination coefficient R^2^ was used for evaluation of the internal validation. R^2^ reflects the goodness of fit, while Q^2^ reflects the predictive ability of the model. The 7-fold leave out procedure (default setting in SIMCA 15.0.2) was used for Q^2^ measurements. R^2^X is the amount of variation in X that is uncorrelated to Y with systematic variation. It shows whether data can be well interpreted. R^2^X(cum) is the total sum of variation in X that is uncorrelated to Y. R^2^Y is the proportion of the variance of the response variable that is explained by the model. R^2^Y(cum) is the total sum of variation in Y explained by the model. Q^2^(cum) reflects the goodness of prediction calculated by full cross validation. R*^2^*X*,* R*^2^*Y, and Q*^2^*values (not less than 0.5) recommended a powerful model with predictive reliability [53]. R^2^X(cum) and Q^2^(cum) values must be less than 0.2–0.3 [54,55]. In addition, misclassification tables were produced for the constructed models, as well as a permutation test (repeated 100 times) for the overall model. Referring to the latter, the criteria for validity of both R^2^ (original model) and Q^2^ (predictive model), located to the right, and permutated R^2^ (original model) and Q^2^ (predictive model), located to the left, and all Q^2^ values to the left and right, are lower than the original R^2^ values.

## 3. Results and Discussion

### 3.1. Stable Isotope Analysis of Οlive Oils

Hydrogen and oxygen isotope composition are generally affected by climatic and environmental conditions [56]. More specifically, factors such as temperature [57], precipitation [58], air humidity, soil and plant evapotranspiration [59], and water stress [60] are related with isotopic composition of hydrogen and oxygen. Moreover, carbon isotope composition is influenced by humidity [61], ground and rain water, temperature [60], sea distance, longitude, and latitude [57]. The climatic parameters of the examined olive oil cultivation areas in Greece according to the data of EMY (the Hellenic National Meteorological Service) are presented in Table 1.

The stable isotope values act as ecophysiological tracers of natural processes with very good discriminatory power [62]. The traceability that can be achieved by measuring stable isotope values is based on the assumption that the isotope values of plants reflect the characteristics of the specific environment [62]. In (Table 2), the mean, standard deviation, minimum, and maximum values of δ^2^H, δ^18^O, and δ^13^C of three monocultivars, i.e., Maurolia (Messinia), Lianolia Kerkyras (Preveza), and Koroneiki (Preveza), thus from two geographic areas, i.e., Messinia and Preveza, are presented.

The stable isotope values of our samples varied between −152.1 and 23.4. Carbon isotope values varied between −31.4 and −27.8, hydrogen isotope values varied between −152.1 and −130.4, and oxygen isotope values varied between 16.7 and 23.4. It is important to note that both cultivars from Preveza (i.e., Lianolia Kerkyras and Koroneiki) had more similar isotope values compared to the Maurolia cultivar from Messinia, as shown in Table 2. Relatively lower values of oxygen isotopes are correlated with areas of high elevation, inland location, and cool climate, while higher values are associated with low elevation, coastal location, and warmer climate [62]. The very specific climatic data that differentiates the two areas from which the samples were taken refers to rainfall, with Preveza having higher values of precipitation as presented in (Table 1). This is related to the lower values of carbon isotopes in Preveza compared to Messinia. In general, samples from the southern region (Messinia) had higher values for carbon and hydrogen isotopes compared to the northern region (Preveza). Moreover, the oxygen isotope ratio reflects water-related processes in plants. Comparing the climatic data (Table 1) with the isotopic composition of examined samples (Table 2), cv. Lianolia Kerkyras and cv. Koroneiki, both from Preveza, had higher oxygen isotope compositions due to higher values of precipitation.

### 3.2. Chemometric Discrimination of Olive Oils

**Figure 1:**
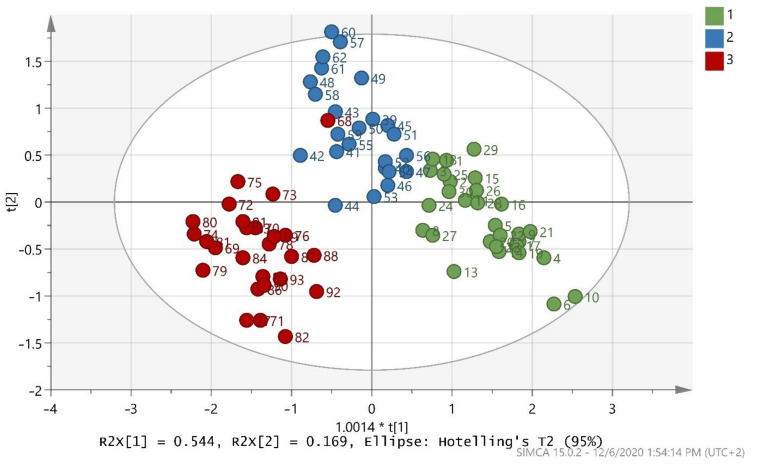
Score scatter plot (t2/t1) from OPLS-DA modeling of the training set where 1 = Maurolia (Messinia), 2 = Lianolia Kerkyras (Preveza), and 3 = Koroneiki (Preveza). A = 2 + 1 components, R^2^X(cum) = 0.998, R^2^Y(cum) = 0.723, and Q^2^(cum) = 0.708 and 1.0014 * t[1] means 1.0014 × t[1].

The training set in Figure 1 indicates that good discrimination of the samples in the three classes was achieved. Difference of microclimate and soil could be a possible explanation for the dispersal of samples of the same cultivar [63]. The validation values R^2^X(cum) = 0.998, R^2^Y(cum) = 0.723, and Q^2^(cum) = 0.708 showed a good fit and prediction ability of the training set. Moreover, the misclassification table for the OPLS-DA modeling is presented in Table 3, and it also showed 93.75% correct classification of the 80 samples in the three classes regarding cultivar. A low Fischer value of *p* < 0.05 emphasized the statistical importance of the training set. In more detail, Maurolia cultivar samples (class 1) were all correctly classified (100% correct classification), but Lianolia Kerkyras (class 2) and Koroneiki (class 3) cultivar samples reduced their class’ correct classification to 83.33 and 96.15%, respectively. Classes 2 and 3, both originating from Preveza, were well separated.

It is important to note that sample 68, which was labelled as cv. Koroneiki from Preveza (class 3), fell into cv. Lianolia Kerkyras from Preveza (class 2). The first reason for this discrepancy is that a labeling mistake might have occurred during sampling. It is very common in fields for the majority of olive trees to have one main variety with some other varieties scattered amongst them. Particularly, in our case, cv. Lianolia Kerkyras and cv. Koroneiki were harvested at the same time in the Preveza area. The outliers found in this study are justified by the fact that the specific samples were indeed blended (i.e., from olive fruits that came from both varieties due to co-cultivation in the same field). Co-cultivation essentially reinforces the fact that this model is capable of detecting samples that are not pure cv. Lianolia Kerkyras or pure cv. Koroneiki, thus not purely monovarietal. There are oils sold in the market with an indication of monovarietal, but since this is not always the truth, this indication clearly has an economic impact. Monovarietal olive oils are more expensive, and so this is consumer deception, which points to fraud. The model presented here can detect this type of adulteration and consequently detect an important type of fraud. The second reason is due to sample contamination during olive oil extraction in the olive mill. Contamination may be related to impurities, such as traces of different cultivar residues.

**Figure 2:**
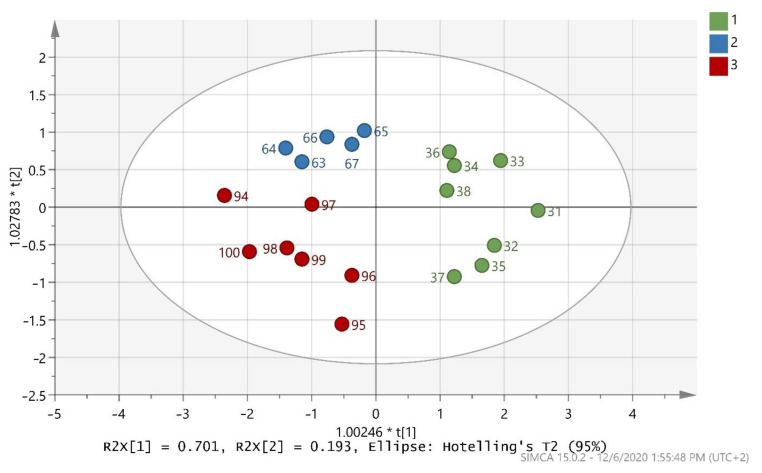
Score scatter plot (t2/t1) from OPLS-DA modeling of the test set where 1 = Maurolia (Messinia), 2 = Lianolia Kerkyras (Preveza), and 3 = Koroneiki (Preveza). A = 2 + 0 + 0 components, R^2^X(cum) = 0.894, R^2^Y(cum) = 0.706, and Q^2^(cum) = 0.641 and 1.00246 * t[1] means 1.00246 × t[1] as well as 1.02783 * t[2]] means 1.02783 × t[2]].

The test set in (Figure 2) indicates that the 20 randomly selected samples from the three classes were able to present a very good classification. All 20 samples were 100% correctly classified in the three classes, as also shown by (Table 4). R^2^X(cum) = 0.894, R^2^Y(cum) = 0.706, and Q^2^(cum) = 0.641 were acceptable and the test set was valid.

**Figure 3:**
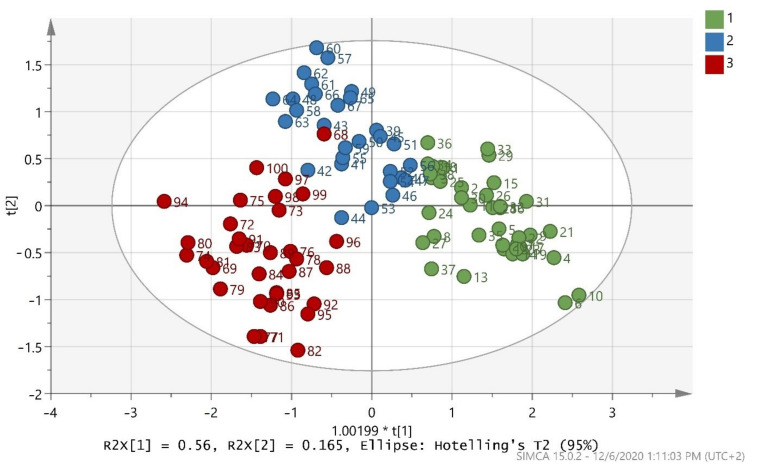
Score scatter plot (t2/t1) from OPLS-DA modeling of the overall model (whole database) where 1 = Maurolia (Messinia), 2 = Lianolia Kerkyras (Preveza), and 3 = Koroneiki (Preveza). A = 2 + 1 + 0 components, R^2^X(cum) = 0.999, R^2^Y(cum) = 0.695, and Q^2^(cum) = 0.681 and 1.00199 * t[1] means 1.00199 × t[1].

**Figure 4:**
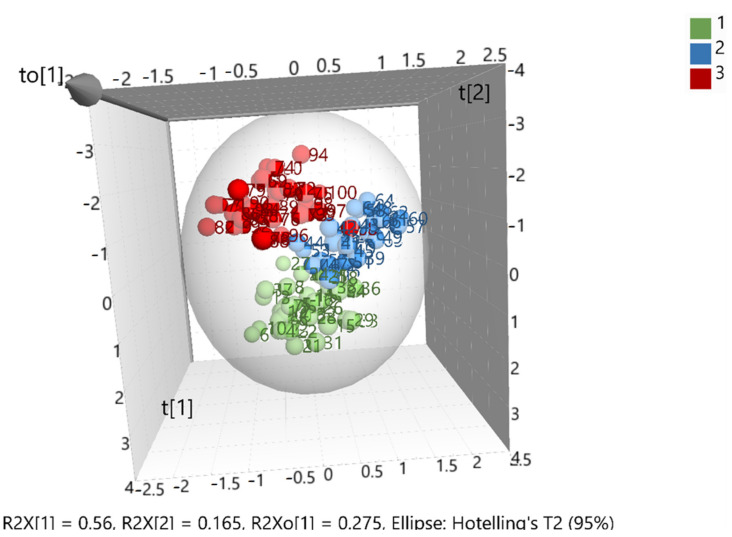
Three-dimensional illustration of the score scatter plot in Figure 3 where 1 = Maurolia (Messinia), 2 = Lianolia Kerkyras (Preveza), and 3 = Koroneiki (Preveza).

**Figure 5:**
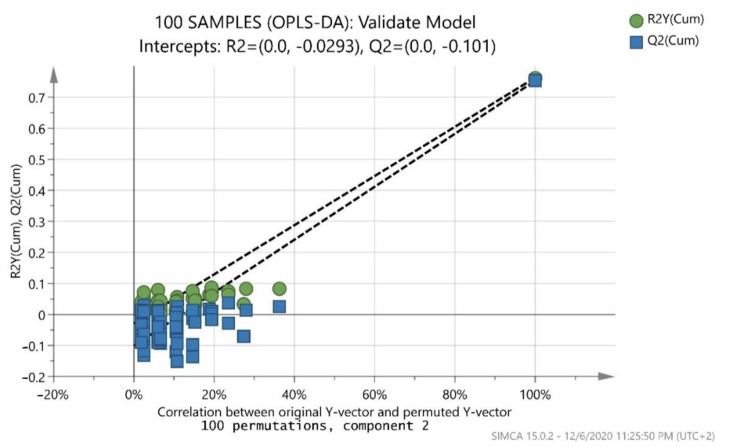
Permutation test of the overall model (whole database) with 100 permutations where both R^2^ (original model) and Q^2^ (predictive model) located at right and permutated R^2^ (original model) and Q^2^ (predictive model) located left.

After merging the training and test sets, the overall model (whole database) was constructed, which is presented in (Figure 3). The validation values R^2^X(cum) = 0.999, R^2^Y(cum) = 0.695, and Q^2^(cum) = 0.681 showed a good fit and prediction ability of the model. In addition, (Figure 4) presents the three-dimensional illustration of the score scatter plot in (Figure 3). Furthermore, misclassification table for the OPLS-DA modeling is presented in (Table 5), and it shows 91% correct classification of the 100 samples in the three classes regarding cultivar. The low Fischer value of *p* < 0.05 emphasized the statistical importance of the model. In more detail, Maurolia (Messinia) class had only 1 sample out of 38 wrongly classified and 97.37% correct classification. Lianolia Kerkyras (Preveza) class gave 75.86% correct classification and it had 7 samples out of 29 whose distance from the center of class 2 was longer than what was expected; further study needs to take place for this class. However, a possible explanation of the low percentage of classification in cv. Lianolia Kerkyras samples (75.86%) could be explained by the fact that those 7 wrongly classified samples (out of 29) could be derived from an olive grove where both cv. Lianolia Kerkyras and cv. Koroneiki olive trees were co-cultivated. From Koroneiki (Preveza) class, only 1 sample out of 33 was wrongly classified, and the class correct classification was 96.97%. In addition, sample 68 was indicated as a misclassified sample in (Figure 3 and Figure 4) as well as in (Figure 1), confirming the robustness of both training and overall sets. Figure 5 shows a random permutation test with 100 permutations used for the validation of goodness of fit and the predictability of these results. The R^2^Y values of all permuted models were lower than the original model’s R^2^Y value (0.695); most of the Q^2^ regression lines showed negative intercepts (0.0−0.101).

Further chemometric analysis showed that O and H stable isotopes were more important variables for the constructed model than C isotopes. Stable isotope ratio analysis variations of ^13^C/^12^C proved to be a useful tool for characterizing samples from different regions with very different climatological and geographic characteristics [26,27,64,65,66,67,68,69] as isotope ratios are affected by latitude, which indicates the distance from the sea, and environmental conditions during the growing of trees (water stress, atmospheric humidity, and temperature) as co-factors of variability. Since all the samples of this study were cultivated in coastal locations, this explains why C stable isotopes were not as important as O and H. Samples were discriminated mainly by olive cultivar and, partially, by geographical origin. This is congruent with the recent study of Alves de Carvalho et al. (2020) [70].

The choice for the study of these varieties was made based on specific parameters. Specifically, the Maurolia variety, along with cv. Koroneiki and cv. Athinolia, are the main varieties grown in southeastern Peloponnese, one of the most important olive oil-producing areas. Compared to the other two varieties, the Maurolia variety matures and is harvested first at the beginning of the olive harvesting period. This is very important, because increased profit could arise from the very first harvest. Moreover, as monovarietal Maurolia olive oil is characterized by balanced qualitative characteristics, manufacturers prefer it in the case that they want produce blends with more bitter or more spicy oils [37]. Although Maurolia olive oil is of high quality, similar to Koroneiki olive oil, [37], this variety has not been included in any catalogue of denominations related to foodstuff authenticity, in contrast to the Koroneiki and Lianolia Kerkyras olive oils, which have been characterized as PDO and PGI varieties, respectively. Instead, Koroneiki cultivar is the most widespread all over the Greece, and the most well-known Greek olive cultivar. It is cultivated in many areas of the country, mainly in Peloponnese, Crete, and in the northwestern part of country [38,71]. Regarding Lianolia Kerkyras, this cultivar is mainly cultivated in the Ionian Islands and in the geographical region of Epirus. As the Koroneiki variety has the greatest reputation both in Greece and abroad, and thus achieves the best prices, it could possibly be a blend of the lesser known and less widespread varieties of Maurolia and Lianolia Kerkyras.

This is the first qualitative attempt to study three important Greek cultivars and extract a robust chemometric model capable of discriminating olive oils based on geographic origin. The qualitative results of this study answer the important question of which geographic area the samples of the three cultivars come from. The method proposed here can be enriched in the future by creating synthetic samples from different geographic and cultivar origins. The big challenge involves discriminating adulterant mixtures of olive oils from the market. By adding the synthetic samples in the present chemometric model, a possible fourth class will be generated. Unknown adulterated samples may be detected in the future, as the adulterant samples will be classified in the fourth class of synthetic samples.

Moreover, a quantitative method is considered necessary for the next attempt, which will be answering the significant question of how much adulteration the unknown samples contain. Those synthetic samples could be mixtures made of different proportions (adulteration levels, %*v*/*v*) of olive oils, as Tsopelas et al. proposed [72]. In the future, the more similar the adulteration level of every unknown adulterated and synthetic sample, the closer the distance between them on a score scatter plot. Subsequently, the adulteration level of the spiked samples will show the adulteration level of every unknown sample in question by observing their positions after classification. Future predictions are important, and this study is the beginning of a bigger model which will be developed in order to classify unknown olive oils from the market.

## 4. Conclusions

By combining IRMS data and OPLS-DA through a multivariate statistical approach, a statistical model able to discriminate olive oils based on geographical origin was obtained with a successful discrimination ability at around 91%. Both δ^18^O and δ^2^H were the most important isotope markers for discrimination of olive oils. The investigation carried out in the present work can be used as a reliable and powerful tool for the characterization and authentication of Greek olive oils. Future determinations of unknown samples can easily occur based on the model depicted here.

## Figures and Tables

**Table 1:** The climatic parameters of the examined olive oil cultivation areas in Greece for the harvest year 2019.

	Precipitation (mm)/Month		Temperature (°C)		Relative Humidity (%)	
	Messinia	Preveza	Messinia	Preveza	Messinia	Preveza
Average	64	91	17	17.2	69	67
Minimum	6	13.4	9.8	8.7	57.7	59.2
Maximum	141.7	199.8	26.5	26.5	75	74.1

**Table 2:** Stable isotope ratios (^13^C/^12^C, ^2^H/^1^H, ^18^O/^16^O) of Greek olive oils from three monocultivars, i.e., Maurolia (Messinia), Lianolia Kerkyras (Preveza), and Koroneiki (Preveza), thus from two geographic areas, i.e., Messinia and Preveza.

Cultivar (Area)	Stable Isotopes	Mean	Standard Deviation	Minimum	Maximum
Maurolia (Messinia)	δ^2^H/‰	−134.7	2.4	−140.4	−130.4
	δ^13^C/‰	−29.3	0.5	−30.4	−28.4
	δ^18^O/‰	19.7	1.1	16.7	21.4
Lianolia Kerkyras (Preveza)	δ^2^H/‰	−138.4	1.9	−141.7	−135.8
	δ^13^C/‰	−30.1	0.5	−30.8	−29.2
	δ^18^O/‰	21.8	0.6	20.0	22.4
Koroneiki (Preveza)	δ^2^H/‰	−137.4	3.3	−152.1	−139.2
	δ^13^C/‰	−29.7	0.7	−31.4	−27.8
	δ^18^O/‰	21.7	0.8	20.2	23.4

**Table 3:** Misclassification table from OPLS-DA modeling of the training set, where 1 = Maurolia (Messinia), 2 = Lianolia Kerkyras (Preveza), and 3 = Koroneiki (Preveza).

	Members	Correct	1	2	3
1	30	100%	30	0	0
2	24	83.33%	3	20	1
3	26	96.15%	0	1	25
Total	80	93.75%	33	21	26
Fisher’s prob.	4.3 × 10^−11^				

**Table 4:** Misclassification table from OPLS-DA modeling of the test set where 1 = Maurolia (Messinia), 2 = Lianolia Kerkyras (Preveza), and 3 = Koroneiki (Preveza).

	Members	Correct	1	2	3
1	8	100%	8	0	0
2	5	100%	0	5	0
3	7	100%	0	0	7
Total	20	100%	8	5	7
Fisher’s prob.	1 × 10^−8^				

**Table 5:** Misclassification table from OPLS-DA modeling of the overall model (whole database) where 1 = Maurolia (Messinia), 2 = Lianolia Kerkyras (Preveza), and 3 = Koroneiki (Preveza).

	Members	Correct	1	2	3
1	38	97.37%	37	1	0
2	29	75.86%	6	22	1
3	33	96.97%	0	1	32
Total	100	91%	43	24	33
Fisher’s prob.	2.3 × 10^−8^				

## Data Availability

Not applicable.
